# Outcomes in a diabetic population of south Asians and whites following hospitalization for acute myocardial infarction: a retrospective cohort study

**DOI:** 10.1186/1475-2840-9-4

**Published:** 2010-01-22

**Authors:** Aman PK Nijjar, Hong Wang, Kaberi Dasgupta, Doreen M Rabi, Hude Quan, Nadia A Khan

**Affiliations:** 1 Department of Medicine, University of British Columbia, BC, Canada; 2 Center for Health Evaluation and Outcomes Sciences, University of British Columbia, BC, Canada; 3 Department of Medicine, McGill University, Quebec, Canada; 4 Departments of Medicine, Community Health and Cardiac Sciences, University of Calgary, AB, Canada; 5 Department of Community Health Sciences, University of Calgary, AB, Canada

## Abstract

**Background:**

The aim of this study was to determine whether South Asian patients with diabetes have a worse prognosis following hospitalization for acute myocardial infarction (AMI) compared with their White counterparts. We measured the risk of developing a composite cardiovascular outcome of recurrent AMI, congestive heart failure (CHF) requiring hospitalization, or death, in these two groups.

**Methods:**

Using hospital administrative data, we performed a retrospective cohort study of 41,615 patients with an incident AMI in British Columbia and the Calgary Health Region between April 1, 1995, and March 31, 2002. South Asian ethnicity was determined using validated surname analysis. Baseline demographic characteristics and co-morbidities were included in Cox proportional hazard models to compare time to reaching the composite outcome and its individual components.

**Results:**

Among the AMI cohort, 29.7% of South Asian patients and 17.6% of White patients were identified as having diabetes (n = 7416). There was no significant difference in risk of developing the composite cardiovascular outcome (Hazard Ratio = 0.90, 95% CI = 0.80-1.01). However, South Asian patients had significantly lower mortality at long term follow-up (HR = 0.62, 95% CI = 0.51-0.74) compared to their White counterparts.

**Conclusions:**

Following hospitalization for AMI, South Asian patients with diabetes do not have a significantly different long term risk of a composite cardiovascular outcome compared to White patients with diabetes. While previous research has suggested worse cardiovascular outcomes in the South Asian population, we found lower long-term mortality among South Asians with diabetes following AMI.

## Background

Patients with Type 2 diabetes are at increased risk of cardiovascular morbidity and mortality. Incidence of initial myocardial infarction has been shown to be much higher in individuals with diabetes relative to their non-diabetic counterparts [1]. Mortality from heart disease is 2-4 times greater in adults with diabetes [2]. The incidence of congestive heart failure is 2.5 times the rate of patients without diabetes. That rate increases to over 10 times in patients under the age of 45 [3].

South Asian patients have a higher prevalence of cardiovascular disease and cardiovascular mortality compared to other ethnic groups such as people of Chinese or European ancestry [4]. One of the primary factors thought to explain this higher prevalence is the increased prevalence of diabetes among South Asian patients [5,6]. Overall, diabetes in South Asian patients is uniformly more prevalent than in White populations [5]. Moreover, when only examining individuals with diabetes, some studies still suggest elevated mortality risk among South Asians [7]. Some authors have found that diabetes and other conventional risk factors may not be sufficient to account for the excess cardiovascular mortality exhibited in South Asians [8]. One possibility is that the diabetes in South Asian patients is of a more aggressive variety than in White populations, leading to increased cardiovascular complications following acute myocardial infarction (AMI).

We sought to clarify the risk increase associated with diabetes among South Asian and White patients following hospitalization for an incident AMI. Specifically, we conducted a retrospective cohort study of persons with AMI and investigated a composite primary outcome of mortality, recurrent AMI, and congestive heart failure (CHF) requiring hospitalization. Outcomes were compared between South Asian and White populations and with and without the presence of diabetes. Secondary outcomes were the individual components of the composite outcome: short-term mortality (≤ 30 days following index AMI), long-term mortality (> 30 days), recurrent AMI, and CHF requiring hospitalization.

## Methods

Data for this study were obtained from hospital administrative discharge records of all hospital institutions in British Columbia (BC) and the Calgary Health Region Area (April 1, 1994 to March 31, 2003). Hospital administrative data contain up to 25 diagnoses for each hospital admission. Index cases of AMI were identified using validated International Classification of Diseases, 9^th ^Revision (ICD-9) and 10^th ^Revision (ICD-10) coding algorithms (410.× and 121.×, respectively). Patients with a baseline history of diabetes at the time of diagnosis of AMI were identified using the codes 250.× (ICD-9) and E10.0-14.9 (ICD-10). Other co-morbid illnesses, socio-demographic data, and outcomes of AMI recurrence and hospitalization for CHF were extracted from hospital administrative data. Mortality data were obtained from the BC Vital Statistics File and the Alberta Registry File.

To further characterize the severity of cardiac disease in a subset of the cases, data on coronary anatomy were obtained from the BC Cardiac Registry (data provided by the Provincial Health Services Authority of BC) and APPROACH cardiac registry files from January 1999 to December 2002. Cardiac catheterization results were classified into six categories, namely disease affecting: 1) single vessel, 2) two vessels, 3) three vessels, 4) left main coronary artery, 5) three vessels including 95% of the proximal left anterior descending (LAD) coronary artery, or 6) normal coronary anatomy. All invasive cardiac procedures performed in BC and Alberta are recorded in these registries with accompanying details on type of procedure and results.

Ethnicity and residence status was determined using population registries that contain data on all registered persons within each province. Data from the different sources were linked via personal health numbers, a 10-digit unique identifier for health care services that is assigned to every Canadian resident.

### Inclusion and Exclusion Criteria

AMI identification was through validated algorithms using ICD-9 and ICD-10 codes [9]. Index cases of AMI were limited to those occurring between April 1, 1995 and March 31, 2002. Patients were excluded if they were less than 20 years old, had a previous AMI diagnosis in the year prior to their admission, and a hospital stay of less than one day. These criteria, except hospital stays of less than one day, are in line with the diagnostic criteria for identifying AMI patients from hospital discharge administrative data set out by the Canadian Cardiovascular Outcomes Research Team and Canadian Cardiovascular Society [10]. Excluding patients with stays of less than one day was instituted in order to avoid patients who were admitted for one-day cardiac procedures. Patients were also excluded if they were non-residents of the province.

### Study Variables

Variables of co-morbid illnesses were identified through ICD-9 and ICD-10 coding algorithms. Diabetes and co-morbidities in an AMI cohort have been validated for both ICD-9 and ICD-10 algorithms [11]. During the fiscal years of 1995-2000, ICD-9 coding was exclusively used, thereafter ICD-10 codes were employed. Previous validation shows that in the example for diabetes, the ICD-9 coding algorithm had a high level of accuracy when compared with chart review of 193 patients. Overall sensitivity for ICD-9 was 80%, specificity was 98.3%, PPV was 80%, and NPV was 98.3%. In the same example, ICD-10 coding algorithm demonstrated a sensitivity of 66.7%, specificity of 98.9%, PPV of 83.3%, and an NPV of 97.2% [11]. The other co-morbid conditions, based on the Ontario acute myocardial infarction mortality prediction rules, included cardiogenic shock, CHF, cancer, cerebrovascular disease, pulmonary disease, renal failure, cardiac dysrhythmias, hypertension, peripheral artery disease, history of ischemic heart disease, cardiac arrest, and liver disease [12].

Ethnicity information was not part of the hospital administrative data set for the AMI cohort. Instead, surname analysis allowed us to separate patients into the category South Asian (ancestry from India, Pakistan, or Bangladesh) or Chinese (ancestry from China, Taiwan, or Hong Kong). Surname analysis was conducted by merging provincial population registries with the Nam Pehchan computer program [13] and Quan's Chinese name list [14] to define South Asian and Chinese ethnicity, respectively. Nam Pehchan's program has previously been demonstrated to recognize South Asian surnames with a sensitivity of 90-94% and positive predictive value of 63-96% [15]. Furthermore, in the process of validating the Nam Pehchan program, visual inspection by committee found only 0.05% of the 356,555 names examined to have mixed components of South Asian and non-South Asian origins [15]. To create the White patient group, we included all remaining patients who were not categorized as South Asian or Chinese based on surname analysis. While the group classified as "White" contained persons of non-European descent, the overall number was likely to be quite small. Persons considered part of the visible minority that are neither Chinese nor South Asian, represent only 6.67% of BC's entire population and 5.67% of Alberta's population [16]. For the purposes of this study, the 946 Chinese patients (2.3% of the entire cohort) were excluded from analysis.

Our measure of socioeconomic status (SES) was residential postal code level median household income. In order to control for regional differences in access to care, we included a variable of distance to the nearest hospital, calculated between the center of the residential postal code to the nearest hospital. Revascularization capability was also determined for each admitting hospital.

In addition to identifying patients with diabetes through ICD-9 and ICD-10 coding, we ascertained whether a subset of patients was receiving medical therapy for diabetes. We included prescription drug data for individuals over the age of 65. Medications in older adults are funded through PharmaCare, a drug program that assists eligible residents with prescription drug costs. The PharmaCare database has extractable prescription information and drug identification numbers (DIN) were used to detect medications for the treatment of diabetes (insulins or oral hypoglycemic agents). Only patients with prescriptions filled prior to the date of their index AMI were considered in the analysis. This was done in order to exclude individuals diagnosed with diabetes after their AMI hospitalization.

### Statistical Analysis

The AMI cohort was stratified into categories of patients with and without diabetes and these groups were further subdivided into South Asian and White patients. The four subgroups were then characterized by clinical, demographic, and socioeconomic variables.

Crude rates of a composite outcome of mortality, recurrent AMI, and CHF requiring hospitalization were compared using chi-square analysis. Cox's proportional hazard models were then constructed for all outcomes and adjusted for the following: age (≥ 65 years), sex, income quintile, distance to the nearest hospital (≥ 50 km), admission year, admission to a revascularization hospital, and province. The co-morbid conditions, based on the Ontario acute myocardial infarction mortality prediction rules, were also included in the model [12]. We used analysis of deviance residuals to test for possible violations of the proportional hazards assumption for all Cox models. Analyses were performed with SAS statistical software version 9.1 (SAS Institute Inc, Cary, North Carolina).

## Results

We identified a total of 40,669 persons with AMI during the study period. Index cases of AMI were followed for up to eight years (mean of 3.5 years) after their initial admission. There were 2190 South Asian (5.4%) and 38,479 White (94.6%) patients. Among the entire cohort, 7416 (18.2%) were coded as having diabetes and these patients were further subdivided by ethnicity (Table 1). Diabetes was more common among South Asian patients at a prevalence of 29.7% compared to 17.6% of White patients. South Asians with diabetes were younger, lived closer to a hospital, and occupied the lower quintiles of socio-economic status (Table 1). While there were significantly more South Asians with diabetes, they generally tended to have similar rates of other co-morbidities.

**Table 1:** Baseline Characteristics of AMI Cohort

	Diabetes (n = 7416)		Non-Diabetes (n = 33253)	
	**South Asian (%)** **n = 651 (29.7)**	**White (%)** **n = 6765 (17.6)**	**South Asian (%)** **n = 1539 (70.3)**	**White (%)** **n = 31714 (82.4)**

Women	239 (36.7)	2603 (38.5)	380 (24.7)	10156 (32.0)

Age ≥ 65 years	350 (53.8)	4603 (68.0)	778 (50.6)	19648 (62.0)

Distance > 50 km†	76 (11.7)	1983 (29.3)	208 (13.5)	10372 (32.7)

Income-1^st ^quintile∞	162 (24.9)	1473 (21.8)	355 (23.1)	6325 (19.9)

Income-2^nd ^quintile	171 (26.3)	1335 (19.7)	405 (26.3)	5976 (18.8)

Income-3^rd ^quintile	115 (17.7)	1272 (18.8)	302 (19.6)	5812 (18.3)

Income-4^th ^quintile	111 (17.1)	1226 (18.1)	227 (14.7)	6012 (19.0)

Income-5^th ^quintile	80 (12.3)	1147 (17.0)	210 (13.6)	6047 (19.1)

Heart Failure	180 (27.6)	1942 (28.7)	261 (17.0)	5115 (16.1)

Hypertension	271 (41.6)	2712 (40.1)	399 (25.9)	7257 (22.9)

Renal Failure	59 (9.1)	442 (6.5)	46 (3.0)	752 (2.4)

Peripheral Vascular Disease	19 (2.9)	453 (6.7)	21 (1.4)	1133 (3.6)

Cardiogenic Shock	16 (2.5)	160 (2.4)	25 (1.6)	486 (1.5)

Pulmonary Disease	3 (0.5)	57 (0.8)	9 (0.6)	143 (0.5)

Cerebrovascular Disease	19 (2.9)	305 (4.5)	25 (1.6)	815 (2.6)

Dysrhythmia	83 (12.7)	1040 (15.4)	197 (12.8)	4863 (15.3)

Cardiac arrest	17 (2.6)	93 (1.4)	41 (2.7)	559 (1.8)

* Percentages are calculated within each ethnicity

† Calculated distance from patient's home to nearest hospital

∞ Income percentages do not equal 100% due to missing data. Income quintiles are in order of lowest to highest.

We evaluated coronary anatomy data from the respective cardiac registry files of BC and Alberta. Cardiac catheterization was performed on a total of 2235 patients with diabetes and 9452 patients without diabetes (i.e., 28% of the total cohort). Among the patients with diabetes who received cardiac catheterization, there were only significant differences detected among the patients with single vessel disease, with 45 South Asian patients (20%) in this category, compared to 281 (13.5%) White patients (p-value = 0.004). South Asians with diabetes were not more likely to have more severe coronary disease. Furthermore, there were no significant differences between the two ethnic groups in terms of left ventricular function.

Pharmacologic data were available for 4978 patients with diabetes, aged 65 and over. Of the 363 South Asian patients with diabetes, 56 (15.4%) were on no diabetes medications, 240 (66.1%) were on oral glucose-lowering medication, and 67 (18.5%) taking insulin. Among White patients with diabetes, 1104 (23.9%) were on no diabetes medications, 2557 (55.4%) on oral glucose-lowering medication, and 954 (20.7%) taking insulin. South Asian patients were more likely to be taking oral medications whereas White patients were more likely to be on no medications (*p = 0.0002*). There were no differences in the proportion of South Asian and White patients using insulin.

### Outcomes

In unadjusted analyses, the composite outcome and its components of mortality and recurrent AMI were significantly different between South Asians and Whites. Among those with diabetes, South Asian patients were 20% more likely to experience a recurrent AMI but White patients were 11% more likely to reach the composite outcome and 42% more likely to die. Occurrence of CHF did not differ significantly between South Asian and White patients (Table 2).

**Table 2:** Unadjusted outcomes according to diabetes status and ethnicity

	Diabetes			Non-Diabetes		
	**South Asian (%)**	**White (%)**	**p-value**	**South Asian (%)**	**White (%)**	**p-value**

Composite	344 (52.8)	3947 (58.3)	0.01	636 (41.3)	14673 (46.3)	0.0001

Mortality	186 (28.6)	2755 (40.7)	< 0.0001	294 (19.1)	8678 (27.4)	< 0.0001

Recurrent AMI	185 (28.4)	1563 (23.1)	0.002	373 (24.2)	6873 (21.7)	0.02

CHF	115 (17.7)	1116 (16.5)	0.44	121 (7.9)	2759 (8.7)	0.25

*Composite outcome consists of death, recurrent AMI, or congestive heart failure requiring hospitalization

Within both ethnic groups, the presence of diabetes was associated with a higher risk of developing the composite outcome. Compared to South Asian patients without diabetes, unadjusted analyses showed those with diabetes were 28% more likely to reach the composite outcome and 50% more likely to die. Results among White patients showed similar trends with those of South Asian patients (data not shown).

Table 3 shows hazard ratios (HR), adjusted for baseline characteristics, for South Asian compared to White patients. Among patients with diabetes, there was no significant difference in reaching the composite outcome. The risk of long-term mortality was decreased by over a third in South Asian patients with diabetes (HR = 0.62, 95% CI = 0.51-0.74). The other components of the composite outcome showed no significant differences between ethnic groups.

**Table 3:** Time to event for outcome: Adjusted Hazard Ratios compared to Whites*

	Diabetes		Non-Diabetes	
	**South Asian (CI)**	**p-value**	**South Asian (CI)**	**p-value**

Composite	0.90 (0.80-1.01)	0.070	0.89 (0.83-0.97)	0.006

30 day mortality	0.77 (0.59-1.01)	0.060	0.96 (0.79-1.16)	0.656

> 30 day mortality	0.62 (0.51-0.74)	< 0.0001	0.65 (0.56-0.76)	< 0.0001

Recurrent AMI	1.17 (1.00-1.36)	0.054	1.06 (0.95-1.18)	0.299

CHF	1.11 (0.91-1.35)	0.298	0.92 (0.77-1.11)	0.403

*Adjusted Cox regression model includes the following variables: sex, age, distance to nearest hospital, admission year, admission to a revascularization hospital, income quintile, province, and the following co-morbid conditions: heart failure, hypertension, renal failure, peripheral artery disease, cardiogenic shock, cancer, pulmonary disease, cerebrovascular disease, dysrhythmia, cardiac arrest, and liver disease.

When examining adjusted regression models within each ethnic group, patients with diabetes had a higher risk of reaching the composite outcome (data not shown). Both South Asian and White patients with diabetes had an HR of 1.25 compared to patients without diabetes (South Asian 95% CI = 1.09-1.43; White 95% CI = 1.21-1.30) for risk of developing the composite outcome. Short-term mortality was not significantly different in South Asian patients with diabetes when compared to their non-diabetic counterparts. However among White patients with diabetes, the HR was 1.11 (95% CI = 1.02-1.21) for short term mortality. In comparing long-term mortality, both South Asians and Whites with diabetes had a higher risk than patients without diabetes. South Asian patients had an HR of 1.44 (95% CI = 1.13-1.83) while White patients' HR was 1.51 (95% CI = 1.43-1.59) for death occurring greater than 30 days after the index admission.

In our subset of patients 65 and over, for whom we had pharmacologic data, we found insulin use correlated with significantly worse outcomes. Ethnic specific analyses were not carried out due to small sample sizes in the South Asian patient group. Overall, patients taking insulin developed our pre-specified outcomes more often than patients with diabetes who were not taking any hypoglycemic treatment. Hazard ratios were as follows (data not shown): Composite outcome--HR = 1.25 (95% CI = 1.13-1.39); Mortality--HR = 1.28 (95% CI = 1.14-1.44); Recurrent AMI--HR = 1.22 (95% CI = 1.02-1.45); and CHF--HR = 1.40 (95% CI = 1.16-1.68). There were no significant differences when comparing oral hypoglycemic treatment to no treatment.

## Discussion

Our results demonstrate that among those with diabetes and incident AMI, South Asian patients were not at a higher risk than White patients for a pre-defined composite outcome that included death, recurrent AMI, and CHF requiring hospitalization. Our study indicates that diabetes appears to confer similar risk increases following hospitalization for AMI in this population, irrespective of ethnicity.

Aside from the primary composite outcome, South Asian patients had significantly lower long-term mortality following AMI than compared to White patients. The mortality findings differ from previous studies reporting equivalent survival [17-21] among South Asians following AMI. None of these studies adjusted or matched for the presence of diabetes. Wilkinson, et al, reported poorer survival among South Asians, but after adjusting for the presence of diabetes, the excessive risk was removed [22]. Most recently, Fischbacher, et al, from Scotland showed improved survival after AMI among South Asians in a cohort of 4.6 million people, while adjusting for hospital admission for diabetes [23]. Their data suggests that the traditionally high rate of cardiovascular mortality in South Asians may be due to a higher incidence of cardiovascular events as opposed to higher case fatality.

Our study varied in several respects to previous work showing equivalent outcomes between South Asians and White populations following AMI. No previous studies used a composite outcome of fatal and non-fatal events. Some studies only examined short-term mortality (< 1 month) or in-hospital mortality [17,20,21]. Most studies were matched case control studies involving patients admitted to the hospital [17,18,21] and may be prone to selection bias.

While South Asian patients did no worse than their White counterparts, as expected, patients with diabetes overall did worse than patients without diabetes. However, the suggestion that diabetes portends a worse prognosis in South Asians is not supported by our study. Although, the previously mentioned studies only included patients with confirmed AMI and did not focus on patients with diabetes, other routine mortality data from the UK found that the presence of diabetes disproportionately increased the risk of ischemic heart disease mortality in South Asians. Chaturvedi, et al, found that individuals with diabetes and of European descent had mortality rates that were 1.5 times that of individuals without diabetes. In South Asians, that number went up to 3 times [7]. Very similar results were found in a general population-based sample of South Asian migrants in the UK by Forouhi, et al. Over 3000 patients were followed for over 15 years (1988-2006) and comparisons made for coronary heart disease mortality. Again, they found that diabetes increased the mortality risk nearly three-fold compared to South Asian patients without diabetes at baseline. In White patients with diabetes, the excess mortality was 1.5-fold [8]. Our results showed an increased risk of long-term mortality of about 1.5 times for both South Asian and White patients, indicating no significant differential impact of diabetes in South Asians, following hospitalization for AMI.

The earlier UK mortality study by Chaturvedi, et al, did have a different methodology in that they examined routine mortality data from 1985-1986, extracting records that listed diabetes anywhere on the death certificate. Their data included out of hospital death, which can be affected by a number of factors not included in either study, including symptom recognition, availability of emergency medical services, and severity of disease. Furthermore, the study used data that is over twenty years old and cardiovascular care has changed substantially from this time period. The more recent population-based study by Forouhi, et al, followed patients prospectively and also measured overall coronary heart disease mortality. While they attempted to control for several conventional risk factors, the excess mortality risk persisted in South Asians. It is possible they may have included patients with a previous history of AMI and therefore placing them at a greater risk for future cardiovascular mortality, whereas our study excluded such individuals.

In our study, almost half of the South Asian patients were under the age of 65. This was in contrast to White patients, in whom only 32% of individuals with diabetes were under the age of 65 at the time their AMI. The younger age at first AMI for South Asian patients has been noted previously [24], but not specifically in patients with diabetes. Since we found that South Asian patients are having their AMI at an earlier age, they may be more likely to come under medical supervision and pursue aggressive risk factor management, therefore decreasing their risk of death in the long term. This is consistent with prospective UK data demonstrating that South Asian patients were more likely to have cardiac procedures and be taking secondary prevention drugs than compared to White patients with similar clinical need [25].

While we found that South Asians with diabetes had lower long-term mortality, they showed a trend for being more likely to experience a recurrent AMI. Since mortality was significantly lower among the South Asian group, we questioned if patients were surviving longer and therefore had a greater probability of experiencing a recurrent AMI. To eliminate this survivor bias, we re-analyzed the data by removing those patients who had died and found that the trend of an increased rate of recurrent AMI in South Asians patients with diabetes disappeared (HR = 1.00, 95% CI = 0.86-1.17). Survivor bias may also play a role in our demonstrated lower mortality among South Asians since only patients diagnosed and admitted with AMI were included in the study. Therefore, we could have missed patients who had more severe disease and died prior to hospital admission.

We included drug utilization data on a small subset of patients. Our results revealed South Asians were more likely to be prescribed diabetes medications than Whites. Whether this reflected more severe diabetes or better control of diabetes among South Asians was not able to be determined. However, it does provide some hypothesis-generating information on differing patterns of drug prescribing and utilization between ethnicities and its impact on diabetes complications.

### Limitations

There are several limitations with this type of study. As this was a retrospective observational study, we were not able to adjust for all relevant covariates. We were unable to include data on risk factors such as diabetes control, duration of diabetes, tobacco use, physical activity, obesity, and dyslipidemia. However, tobacco use has been shown to be less common among certain South Asian communities [26,27] but it may have more than an additive effect with diabetes in the risk of AMI [28]. Rates of tobacco use, along with other risk factors, could in part, explain the increased mortality among Whites. Although we did adjust for age in our multivariate models, there may be some component of relative youth that we are unable to control for that is contributing to the lower mortality among South Asians. Misclassification of ethnicity may have occurred since we employed surname algorithms to determine South Asian ethnicity. While the specificities associated with these algorithms were high, there may still be some erroneous categorization of ethnicity. Furthermore, the group of White patients may consist of some non-White individuals given that all patients without a South Asian or Chinese surname were included in this category. This group of patients is likely to be quite small since the minority population, excluding South Asians and Chinese, is only about 6% of both BC and Alberta's overall population. On the whole, ethnicity misclassification is likely to be non-differential and would tend to underestimate any differences between the study groups. Finally, although our study focused on a large population-based cohort, the overall number of South Asian patients remained relatively small.

## Conclusions

Although some speculate that South Asian patients possess a particularly malignant form of diabetes compared to others, our study shows South Asian patients with diabetes do not have a significantly different prognosis for fatal and non-fatal events following hospitalization for AMI, compared to White patients with diabetes. In fact, South Asians patients with diabetes experienced a significantly lower mortality rate following hospitalization for AMI. Further research is necessary to better characterize the complex factors that are driving these findings.

## Abbreviations

AMI: acute myocardial infarction; CHF: congestive heart failure; BC: British Columbia; SES: socioeconomic status; HR: hazard ratio; CI: confidence interval; PPV: positive predictive value; NPV: negative predictive value

## Competing interests

The authors declare that they have no competing interests.

## Authors' contributions

APKN and NAK designed the original hypothesis. NAK and HQ participated in the design of the study. HW ran all the statistical analyses. APKN drafted the original manuscript. KD, DMR, HQ, and NAK participated in manuscript revisions. All authors read and approved the final manuscript.
